# SARS-CoV-2 Infection in Health Care Workers of Trieste (North-Eastern Italy), 1 October 2020–7 February 2022: Occupational Risk and the Impact of the Omicron Variant

**DOI:** 10.3390/v14081663

**Published:** 2022-07-28

**Authors:** Luca Cegolon, Federico Ronchese, Francesca Ricci, Corrado Negro, Francesca Larese-Filon

**Affiliations:** 1 Department of Medical, Surgical & Health Sciences, University of Trieste, 34127 Trieste, Italy; negro@units.it (C.N.); larese@units.it (F.L.-F.); 2 Public Health Department, University Health Agency Giuliano-Isontina (ASUGI), 34148 Trieste, Italy; 3 Occupational Medicine Unit, University Health Agency Giuliano-Isontina (ASUGI), 34148 Trieste, Italy; federico.ronchese@asugi.sanita.fvg.it (F.R.); francesca.ricci@studenti.units.it (F.R.)

**Keywords:** biological risk, COVID-19, health care-associated infection, health protection measures, job task, occupational risk, SARS-CoV-2, vaccines

## Abstract

Vaccination coverage against COVID-19 among health care workers (HCWs) of the University Health Agency Giuliano-Isontina (ASUGI) of Trieste (North-eastern Italy) by 1 January 2022 was 90.4% with at least one vaccine dose, 84.9% with at least 2 doses, and 75.1% with 3 doses, 98.2% with Comirnaty (Pfizer BioNtech, New York, NY, USA) versus 1.8% with Spikevax (Moderna, Cambridge, MA, USA). From 1 October 2020 to 7 February 2022, 1652 SARS-CoV-2 infections were notified in HCWs of ASUGI Trieste. Although the overall risk of SARS-CoV-2 contagion increased over time, the rate of occupational infections progressively declined, from 42.5% during the second COVID-19 wave to 15.6% in the fifth. Between 1 January–7 February 2022 (a period dominated by the Omicron variant), albeit no COVID-19-associated hospitalizations were recorded in HCWs of ASUGI Trieste, 669 SARS-CoV-2 infections were counted against 367 cases observed from 1 October to 31 December 2020, the 3 months preceding the implementation of the vaccination campaign against COVID-19. Job tasks and health care settings turned out to be the most significant risk factors for SARS-CoV-2 infection. However, the effect of workplace prevailed over job task on the biological risk, with greater rates of SARS-CoV-2 infections observed among HCWs operating in areas with higher levels of circulation of the virus, particularly COVID-19 dedicated units.

## 1. Introduction

The novel coronavirus disease (COVID-19) was declared a pandemic on 11 March 2020 by the World Health Organization [1]. Since the very early phases, the epidemic heavily burdened the physical and psychological well-being of healthcare workers (HCWs), who had to deal with a completely new infectious threat and an overwhelming amount of severely ill patients to manage. Although generally younger—hence with less chance of developing the severe form of the disease—HCWs have been exposed to a higher risk of SARS-CoV-2 infection than the general population, especially those assigned to job tasks entailing direct/close contact with COVID-19 patients [2,3,4,5]. Patient-facing tasks are the most hazardous in terms of occupational risk of SARS-CoV-2 infection in health care settings, whereas non-clinical workers are more likely to become infected outside the worksite. The fatality risk associated with COVID-19 increases linearly with age (particularly > 65 years) and/or in presence of co-morbidities, including diabetes mellitus, obesity, systemic arterial hypertension, chronic obstructive pulmonary disease, cancer, acute kidney injury, cardiovascular disease, and increased D-dimer [6,7].

Although males are more likely to develop the severe form of the disease, occupational COVID-19 among the health care force is reportedly more frequent among females, an epidemiological figure coherent with their higher employment prevalence among HCWs [2,8]. Whilst COVID-19 vaccines proved effective to protect against the severe form of COVID-19, their role to prevent SARS-CoV-2 infection has been largely questioned.

In view of the above, this study aimed to estimate the risk of SARS-CoV-2 infections and associated factors in HCWs of the University Health Agency Giuliano-Isontina (ASUGI) of Trieste (North-eastern Italy) from 1 October 2020 to 7 February 2022.

## 2. Materials and Methods

HCWs of ASUGI Trieste (*N* = 5019), including two major teaching hospitals, primary care services, health districts, public health services, and administrative/management services have been systematically screened for COVID-19 since the start of the pandemic, becoming an ideal population to estimate the infection risk, the associated factors, and the effectiveness of the respective vaccines [9,10,11,12,13,14,15].

Since COVID-19 vaccination was mandatory for all HCWs of ASUGI by Italian law, clinical and non-clinical HCWs unvaccinated against COVID-19 have been suspended from work, whereas those exempted from vaccination for health reasons were re-assigned to job tasks not entailing patient contact. These suspensions and job task reassignments were mainly enforced in Trieste from September/October 2021 onwards. HCWs with only one or two doses of COVID-19 vaccines by 1 January 2022 had at least one previous confirmed infection by SARS-CoV-2.

The surveillance system adopted by the Occupational Medicine Unit of ASUGI Trieste required routine nasopharyngeal swabs for all HCWs with patient contact to detect SARS-CoV-2 infection by real-time polymerase chain reaction (RT-PCR) on a weekly or monthly basis depending on their job task and risk assessment, re-testing in case of symptoms or history of close contact with confirmed COVID-19 cases. In particular, all HCWs employed in department more likely to host COVID-19 patients (COVID-19 units, internal medicine, infectious diseases, A&E, and hematology wards) were considered at high risk for COVID-19 and screened weekly, regardless of the job task. Additionally, HCWs in contact with immunocompromised patients were also considered at high risk for COVID-19 and screened weekly. For all other HCWs, a monthly screening schedule was followed.

The latter surveillance system was extended also to postgraduate specialist medical trainees (*N* = 534), undergraduate medical/nurse students (*N* = 780), blue collars (*N* = 870), and academic staff/tutors (*N* = 72).

Moreover, HCWs were tested on demand in case of symptoms consistent with COVID-19 or close contact with patients or colleagues positive for SARS-CoV-2, as required by Italian law (each day for 5 days following close contact with confirmed COVID-19 cases). Hospital inpatients were routinely tested every 3 days.

Occupational SARS-CoV-2 infections were defined as those developed following exposure to confirmed COVID-19 cases (patients or colleagues) in health care setting, in absence of housemates or household members positive for SARS-CoV-2. By contrast, non-occupational cases were defined as health care associated SARS-CoV-2 infections without previous exposure to confirmed COVID-19 cases among colleagues or patients. Re-infections were defined as secondary infections in the same HCW, occurring at least 90 days following a previous SARS-CoV-2 infection.

All HCWs testing positive for SARS-CoV-2 were interviewed over the phone for contact tracing, following international guidelines [14]. A structured questionnaire (Supplementary File S1) was used to investigate some demographic information (sex and age), job task, health care worksite, COVID-19 vaccination status (number of doses, date of vaccination), date of SARS-CoV-2 positive swab test, and any COVID-19 associated symptoms of the HCW testing positive for SARS-CoV-2. Additionally, in order to distinguish infections of occupational origin, the HCW was asked whether in the 14 days before testing positive for SARS-CoV-2 had any contact:

- with COVID-19 patients or colleagues positive for SARS-CoV-2 in the workplace; or
- with close friends, housemates, or household members positive for SARS-CoV-2 outside the workplace.

### Statistical Analysis

Quantitative variables were expressed as mean ± standard deviation; categorical data were reported as frequencies and percentages. Differences between continuous data and categorical data were estimated by T-Student and chi-square test, respectively. Risk factors of occupational infections were investigated by multivariable logistic regression, selecting terms significant at univariable analysis. Results were expressed as odds ratio unadjusted (OR) as well as adjusted (aOR), with 95% confidence intervals (95%CI).

Descriptive statistics on vaccination coverage were restricted to 5019 HCWs employed by ASUGI Trieste. Logistic regression analysis on risk of occupational SARS-CoV-2 infections included also staff not employed by ASUGI Trieste, hence undergraduate medical/nursing students, post-graduate medical trainees, blue collars, and academic staff/tutors (total *N* = 7241; Table 1).

STATA 14.2 (StataCorp LLC, College Station, TX, USA) was employed for the analysis.

## 3. Results

**Figure 1:**
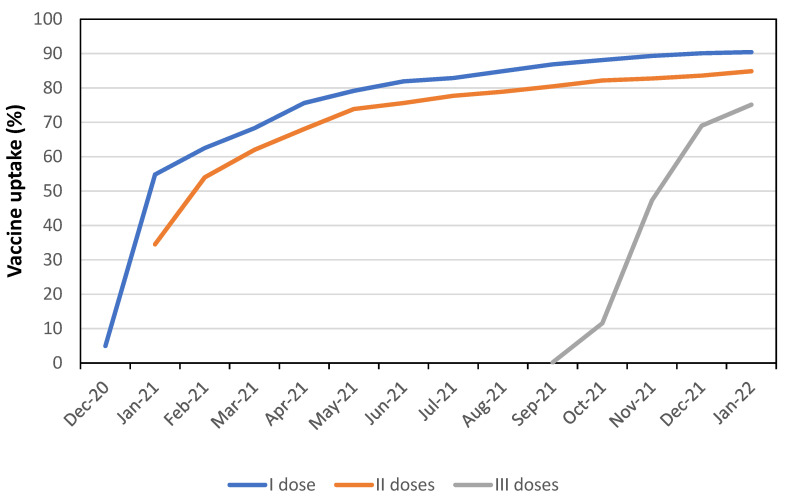
COVID-19 vaccine uptake (%) over time in health care workers (*N* = 5019) employed by the University Health Agency Giuliano-Isontina (ASUGI) of Trieste, by number of doses received.

Our study analyzed the risk of SARS-CoV-2 infection in a population of individuals almost fully vaccinated with m-RNA vaccines (98.2% Comirnaty vs. 1.8 % Spikevax). In Italy, the vaccination campaign against COVID-19 started on 27 December 2020. Vaccine coverage against COVID-19 among 5019 HCWs employed at ASUGI Trieste by 1 January 2022 was 90.4% (=4538/5019) with at least one dose, 84.9% (=4529/5019) with at least 2 doses and 75.1% (=3770/5019) with 3 (Figure 1).

As can be seen from Table 1, a total number of 1652 SARS-CoV-2 infections were notified during the entire study period (1 October 2020 to 7 February 2022) among HCWs of ASUGI Trieste.

During the entire study period (1 October 2020–7 February 2022), 129,335 swab tests were totally performed in the study population.

**Figure 2:**
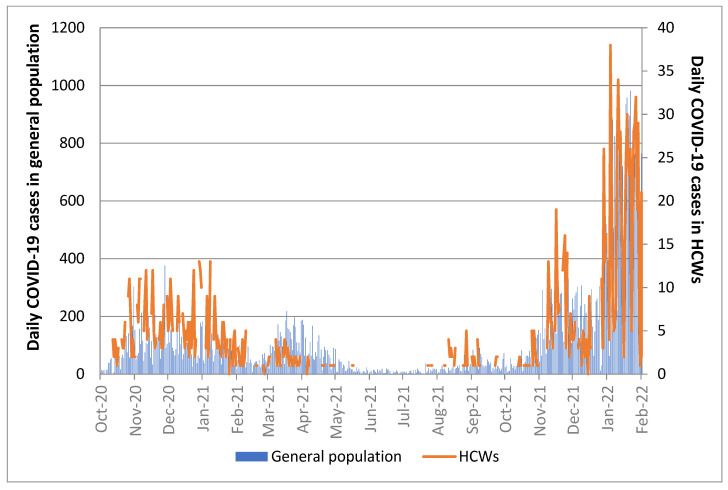
Number of daily SARS-CoV-2 infections in the general population of Trieste versus health care workers (HCWs) of ASUGI Trieste over time (1 October 2020–7 February 2022).

The trend of infections in the study population mirrored the temporal distribution of incident cases in the general population of Trieste during the same time-period (Figure 2).

Between 1 January–7 February 2022 (a period dominated by the Omicron variant), albeit no COVID-19 associated hospitalizations were recorded in HCWs of ASUGI Trieste, 669 SARS-CoV-2 infections were counted against 367 notified from 1 October to 31 December 2020, the 3 months preceding the implementation of the COVID-19 vaccination campaign in Italy (Table 1). Out of 670 total SARS-CoV-2 infections notified during 1 January–7 February 2022, 598 (89.3%) occurred among those who had received at least 2 doses of COVID-19 vaccines (data not shown).

**Figure 3:**
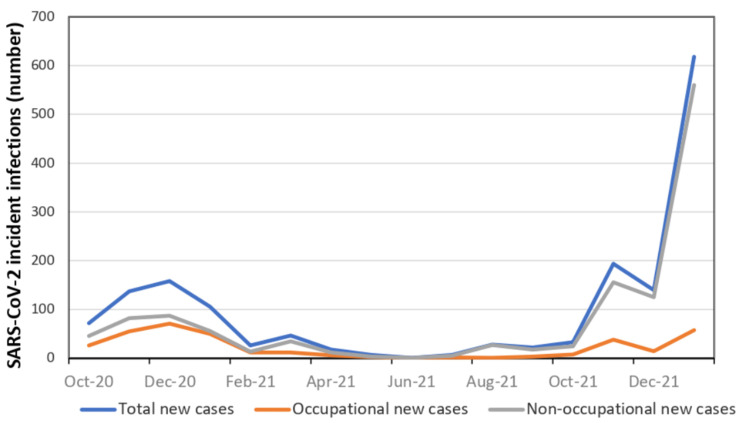
Daily SARS-CoV-2 incident infections among health care workers of ASUGI Trieste (1 October 2020–7 February 2022).

Table 2 shows the descriptive distribution of infections by occupational versus non-occupational origin. As can be noted, the mean age of HCWs was almost identical between occupational (43.7 years) versus non-occupational (43.5 years) infections. Females accounted for 67% SARS-CoV-2 infections and were also infected more frequently (68.9%) than males outside work. Figure 3 illustrates the distribution over time of SARS-CoV-2 infections in HCWs of ASUGI Trieste, by occupational origin. As can be appreciated from Table 2 and Figure 3, although the overall risk of SARS-CoV-2 contagion increased over time, the rate of occupational infections progressively declined, from 42.5% in the second COVID-19 wave to 15.6% during the fifth. After the second wave, the majority of SARS-CoV-2 infections in HCWs of ASUGI Trieste were acquired outside the worksite. The proportion of occupational SARS-CoV-2 infections was particularly higher in medical wards, COVID-19 units and Accident and Emergency (A&E). Considering job tasks, nurses and nurse aides were the categories of HCWs with the highest proportion of occupational SARS-CoV-2 infection.

Table 3 displays the distribution of SARS-CoV-2 secondary infections from 1 April 2021 until 7 February 2022. A total number of 69 SARS-CoV-2 re-infections were recorded during the above time interval. Secondary infections started from November 2021, skyrocketing from January 2022 on. The vast majority of re-infections occurred in females (72.5% = 50/69) and were predominantly of non-occupational origin (84.1% = 58/69).

**Figure 4:**
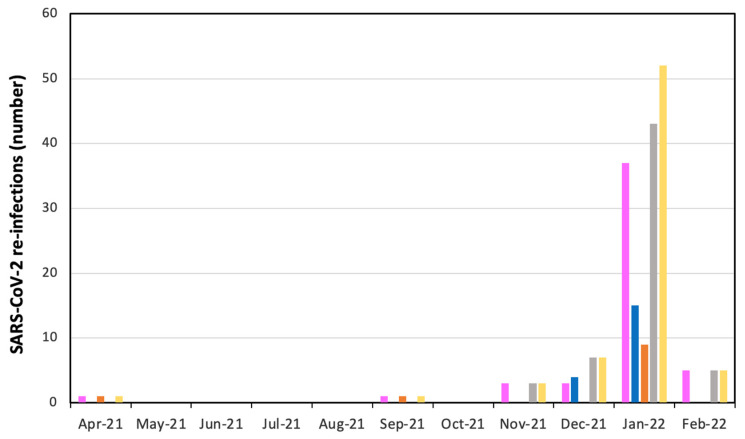
Monthly number of SARS-CoV-2 re-infections in health care workers of ASUGI Trieste (1 April 2021–7 February 2022.

The trend of SARS-CoV-2 re-infections over time from 1 April 2021 until 7 February 2022 can also be visualized in Figure 4.

Table 4 shows the results of univariable as well as of multivariable logistic regression analysis on the entire cohort (*N* = 7241), hence including also external contractors of ASUGI Trieste. Two multivariable logistic model were fitted:

- **Model I**: adjusting for patient’ age; sex; COVID-19 wave, job task; worksite;
- **Model II**: adjusting for COVID-19 wave, job task; worksite

Using the second COVID-19 wave as reference, we identified a decreased trend of occupational risk of SARS-CoV-2 infections in the subsequent waves. Non-clinical workers, typically less exposed to biological risk in the worksite, were used as reference category in the risk assessment by job task and hospital ward. Job task and health care setting turned out to be the most significant risk factors for SARS-CoV-2 infection. As can be seen from multivariable model I, patient-facing tasks were the most hazardous for occupational SARS-CoV-2 infection among HCWs of ASUGI Trieste, especially in COVID-19 dedicated units (OR = 62.21; 95%CI: 10.91; 354.94) and A&E (OR = 132.27; 95%CI: 22.99; 760.98). Health care areas less affected by SARS-CoV-2 infection were those not involving patient contact, as administrative services (OR = 6.67; 95%CI: 1.05; 42.42). Multiple model II (removing age and sex) confirmed the overall results, although the effect size and stratum specific confidence intervals of worksite shrank, whereas the effect size of job task increased, widening also the respective confidence intervals.

## 4. Discussion

### 4.1. Key Findings

During the entire study period (1 October 2020 to 7 February 2022) a total number of 1652 SARS-CoV-2 infections were notified in HCWs of ASUGI Trieste. The effectiveness of m-RNA vaccines seemingly reduced during the fifth COVID_19 wave, with the spread of the Omicron variant. After the second wave, the majority of SARS-CoV-2 infections among HCWs of Trieste occurred outside the workplace, predominantly among females. Patient-facing tasks were the riskiest for occupational SARS-CoV-2 infections, with nurses and nurses’ aides being the professional categories more likely to become infected in the worksite. Nevertheless, the infection risk associated with the workplace prevailed over job tasks. In particular, the risk of contagion increased in A&E and COVID-19 dedicated units, whereas it was lower in health care areas not involving patient contact, such as administrative services.

### 4.2. Interpretation of Findings

Risk reduction rules need to be strictly observed in Italian health care settings in order to benefit from social compensation schemes for sickness absence due to COVID-19. The massive number of SARS-CoV-2 infections among HCWs of ASUGI Trieste during 1 January–7 February 2022 was by far acquired outside health care premises, where health protection measures—such as social distancing, hand washing, and especially the use of personal protection equipment (PPE)—were less stringently enforced. In a survey on 1266 HCWs recruited from a cancer center in Catalunia (Spain) during the first pandemic wave (21 May 2020–26 June 2020), whilst no difference in seroprevalence was observed between onsite workers and teleworkers, living with a person with COVID-19 significantly enhanced the risk of seropositivity to SARS-CoV-2 [16].

Although effective to prevent the severe form of COVID-19, the effectiveness of m-RNA vaccines against the spread of SARS-CoV-2 infection seemingly declined from January 2022 onward (with the spread of the Omicron variant). Human coronaviruses are known to cause respiratory re-infections, regardless of pre-existing humoral immunity [6,17] and Omicron is featured by a higher risk of reinfection than previous variants [18]. In particular, the effectiveness of the current COVID-19 vaccines against the symptomatic disease was reportedly higher for the Delta variant than for Omicron [19]. In the present study compliance with health protection norms was confirmed to be critical to preventing occupational contagion in health care settings, where the lack or suboptimal use of PPE reportedly enhanced the circulation of the virus [14,20].

Following the initial stage of the pandemic, HCWs started to familiarize themselves with PPE [21] and the rate of occupational SARS-CoV-2 infections progressively declined over time in the present study, from 42.5% in the second wave to 15.6% during the fifth (dominated by the Omicron variant). Furthermore, it can be reasonably argued that occupational transmission of SARS-CoV-2 in health care settings might have also occurred during lunch or recreational breaks, social moments when HCWs may inadvertently relax their attention on risk reduction behaviors [22].

Since occupational risk of SARS-CoV-2 infection increases with frequency, duration and intensity of patient contacts, patient-facing tasks inevitably pose the highest risk of contagion. Nurses and nurses’ aides were in fact professional categories more at risk of occupational SARS-CoV-2 infection in the present study. Likewise, in a retrospective study on 2,199,745 individuals recruited from Catalunia (Spain) between 21 March 2020 and 16 September 2021, the age and sex adjusted cumulative rate of SARS-CoV-2 infection was highest in HCWs (27.7%), particularly among nurses’ aides (29.4%), doctors (27.3%) and nurses (26.3%) [23].

However, whilst healthcare settings are among the most hazardous contexts for SARS-CoV-2 infection [16], the present study emphasizes the prevailing effect of the workplace over job tasks. HCWs operating in COVID-19 dedicated units in fact inevitably have the highest level of exposure to the virus, whereas the high rates of infection in A&E might be explained by inadequate compliance with health protection measures, probably due to the emergency working context. Likewise, among the above-mentioned 1266 HCWs from the cancer center in Catalunia, nurses and medical staff working in a COVID-19 dedicated unit exhibited the highest seroprevalence to SARS-CoV-2 as compared to other health care staff [16].

Nevertheless, the very high adjusted risk estimates for occupational SARS-CoV-2 infections associated with worksite were accompanied by very wide confidence intervals, likely reflecting the low numbers involved.

### 4.3. Strengths and Limitations

The present study relies on data from a relatively large population routinely and stringently tested for SARS-CoV-2, thus providing reliable estimates of infection rates. Moreover, the long study period, stretching up to 16 months, enabled to monitor SARS-CoV-2 infections in the same population over time, contrasting the number of infections in the same population before the implementation of the COVID-19 vaccination campaign and after full immunization (by complete vaccination schedule or natural infection). Lastly, this study distinguished occupational versus non-occupational SARS-CoV-2 infections by contact tracing, as recommended by the Italian occupational compensation scheme.

Nevertheless, this study has also some limitations. Contact tracing by questionnaire is intrinsically affected by potential recall bias. Furthermore, some HCWs might have intentionally neglected some sources of SARS-CoV-2 infections outside the workplace (e.g., social events, parties, restaurants, bars, among others) in order to benefit from the occupational compensation scheme, which is more convenient than the non-occupational scheme. However, in the case of coexistent exposures (occupational as well as non-occupational), the approach adopted by the Occupational Medicine Unit in ASUGI Trieste was to consider all these infections as occupational.

A weekly/monthly testing schedule might have skipped some asymptomatic/pre-symptomatic COVID-19 cases, thus potentially underestimating to some extent the true incidence of SARS-CoV-2 infections. However, although endorsed by some authors to monitor SARS-CoV-2 infectious state in HCWs, daily screening by RT-PCR was beyond the capacity of the laboratory service of ASUGI Trieste [24]. Moreover, albeit the UK Health Security Agency recommends 2 rapid swab tests per week for HCWs, to be taken before beginning to work and spaced 3 to 4 days apart [25], the US Center for Disease Prevention and Control (CDC) does recommend to screen fully vaccinated asymptomatic HCWs only if they have been in close contact with a confirmed COVID-19 case [26]. Although difficult to assess the impact of the screening schedule adopted by ASUGI Trieste on the decreasing rate of occupational SARS-CoV-2 infections in HCWs over time, we believe this approach still allowed the early detection of most asymptomatic and pre-symptomatic COVID-19.

Lastly, due to Italian privacy law, out of 481 unvaccinated HCWs the exact number of those suspended from work or exempted from COVID-19 vaccination for health conditions and re-assigned to job tasks not entailing patient contact was not available. However, the latter group contributed to the total number of SARS-CoV-2 notifications during the entire study period, maintaining the original job task until re-assignment. Likewise, suspended HCWs contributed to the number of SARS-CoV-2 infections until work suspension, maintaining their original job task up until then.

## 5. Conclusions

Whilst effective to prevent severe forms of COVID-19, the effectiveness of m-RNA vaccines against the spread of SARS-CoV-2 infection seemingly declined from January 2022 onward, with the fifth wave and the spread of the Omicron variant. Compliance with risk reduction norms was confirmed to be the main preventative factor against SARS-CoV-2 contagion in health care settings. Consistent with the open literature, nurses and nurses’ aides were professional categories more at risk of occupational SARS-CoV-2 infection, stressing the importance of patient-facing contact as a critical risk factor for the contagion. However, the effect of the workplace prevailed over job tasks on the biological risk, with greater rates of SARS-CoV-2 infections observed among HCWs operating in areas with higher levels of exposure to the virus, particularly COVID-19 dedicated units and A&E.

## Figures and Tables

**Table 1:** Distribution of health care staff of ASUGI Trieste by vaccination status and COVID-19 incident cases. Number (N) and percentage (%). HCW = Health care workers. NA = not available.

JOB TASK	STRATA	HCWs		COVID-19 INCIDENT CASES			
		Total N (Col %)	Unvaccinated N (Row %) ^@^	TOTAL		(1 Oct 2020–31 Dec 2020) N (% Total Cases)	(1 Jan 2022–7 Feb 2022) N (% Total Cases)
				N (Col %)	Row % ^@@^		
**CLINICIANS**	**Medical doctors**	900 (12.4)	57 (6.3)	203 (12.3)	22.6	43 (21.2)	65 (32.0)
	**Post graduated trainees**	534 (7.4)	NA	145 (8.8)	27.1	29 (20.0)	99 (68.3)
	**Nurses**	1824 (25.2)	134 (7.3)	485 (29.4)	26.6	126 (26.0)	193 (39.8)
	**Nurse aids**	778 (10.7)	87 (11.2)	191 (11.6)	24.6	48 (25.1)	66 (34.6)
	**Health** technicians *	662 (9.1)	112 (16.9)	190 (11.5)	28.7	28 (14.7)	84 (44.2)
	**Medical/Nursing ** **students**	780 (10.8)	40 (5.1)	129 (7.8)	16.5	30 (23.2)	49 (38.0)
**NON-** **CLINICAL ** **WORKERS**	**Clerks**	821 (11.3)	51 (6.2)	131 (7.9)	16.0	24 (18.3)	51 (38.9)
	Blue collars **	870 (12.0)	NA	159 (9.6)	18.3	37 (23.3)	56 (35.2)
	**Academic staff/tutors ^**	72 (1.0)	NA	19 (1.1)	26.4	2 (10.5)	7 (36.8)
**TOTAL**		**7241**	**481 (6.6)**	**1652 (100)**	**22.8**	**367 (22.2)**	**670 (40.6)**

^@^ = number of unvaccinated HCWs/corresponding stratum specific number of HCWs; ^@@^ = number of SARS-CoV-2 infections/corresponding stratum specific number of HCWs. * Laboratory services; radiology services. ** General cleaners; food workers; gardeners; maintainers; guardians, other. ^ Academic staff/tutors include 34 ASUGI employees and 38 University employees.

**Table 2:** Distribution of SARS-CoV-2 infections among health care workers of Trieste, by occupational origin. Number (N); percentage (%); mean (M) ± standard deviation (SD); *p*-value = chi-square test *p*-value; ^&^ = Mann–Whitney test *p*-value. Tot = total. A&E = Accident and emergency service.

FACTORS	STRATA	OCCUPATIONAL INFECTIONS N (col %)	NON-OCCUPATIONAL INFECTIONS N (col %)	TOTAL INFECTIONS N (col %)	*p*-Value
**TOTAL—N (row %)**		358 (21.7)	1294 (78.3)	1652 (100)	
**Age (years)**	**M ± SD**	43.7 ± 11.6	43.5 ± 11.6	42.8 ± 12.5	0.053 ^&^
**Sex**	**Females**	246 (68.7)	858 (66.3)	1104 (66.8)	0.392
	**Males**	112 (31.3)	436 (33.7)	548 (33.2)	
**COVID-19** **Waves**	**II (1 Oct–31 Dec 2020)**	152 (42.5)	215 (16.6)	367 (22.2)	<0.001
	**III (1 Jan–31 Mar 2021)**	74 (20.7)	104 (8.0)	178 (10.8)	
	**III b (1 Apr–30 Sept 2021)**	14 (3.9)	67 (5.2)	81 (4.9)	
	**IV (1 Oct–31 Dec 2021)**	62 (17.3)	295 (22.8)	357 (21.6)	
	**V (1 Jan–7 Feb)**	56 (15.6)	613 (47.4)	669 (40.5)	
**WORKSITE**					
**Academic staff/tutors**		0	19 (1.5)	19 (1.1)	<0.001
**General services (external workers non-sanitary)**		3 (0.8)	305 (23.6)	308 (18.6)	
**Administrative services**		8 (2.2)	99 (7.6)	107 (6.5)	
**Radiology and other hospital services**		19 (5.3)	143 (11.1)	162 (9.8)	
**Surgical wards**		36 (10.1)	206 (15.9)	242 (14.6)	
Territorial health services */health care management/psychologists		59 (16.5)	247 (19.1)	306 (18.5)	
**Medical wards**		60 (16.8)	167 (12.9)	227 (13.7)	
**COVID-19 hospital units**		90 (25.1)	68 (5.3)	158 (9.6)	
**A&E**		83 (23.2)	40 (3.1)	123 (7.5)	
**JOB TASK**					
**Clinicians**	**Medical doctors** ** (including postgraduate trainees)**	69 (19.3)	279 (21.6)	348 (21.1)	<0.001
	**Nurses**	168 (46.9)	317 (24.5)	485 (29.4)	
	**Nurse aides**	78 (21.8)	113 (8.7)	191 (11.6)	
	**Health technicians ^$^**	28 (7.8)	162 (12.5)	190 (11.5)	
	**Medical/Nursing students**	0 (0.0)	129 (10.0)	129 (7.8)	
**Non-clinical workers**	**Clerks**	12 (3.3)	119 (9.2)	131 (7.9)	
	Blue collars **	3 (0.1)	156 (12.1)	159 (9.6)	
	**Academic staff/tutors**	0	19 (1.5)	19 (1.1)	

* Primary care services; health districts; outpatient; public health department ** General cleaners; food workers; gardeners; maintainers; guardians, other ^$^ Laboratory services; radiology services.

**Table 3:** SARS-CoV-2 re-infections in health care workers of ASUGI Trieste from 1 April 2021 to 7 February 2022. Number (N) and percentage (%).

CALENDAR MONTH	RE-INFECTIONS BY SEX		RE-INFECTIONS BY OCCUPATIONAL ORIGIN		TOTAL RE-INFECTIONS N (%)
	FEMALES N (%)	MALES N (%)	OCCUPATIONAL N (%)	NON-OCCUPATIONAL N (%)	
**April 2021**	1 (2.0)	0	1 (9.1)	0	1
**May 2021**	0	0	0	0	0
**June 2021**	0	0	0	0	0
**July 2021**	0	0	0	0	0
**August 2021**	0	0	0	0	0
**September 2021**	1 (2.0)	0	1 (9.1)	0	1
**October 2021**	0	0	0	0	0
**November 2021**	3 (6.0)	0	0	3 (5.2)	3 (4.3)
**December 2021**	3 (6.0)	4 (21.1)	0	7 (12.1)	7 (10.1)
**January 2022**	37 (74.0)	15 (71.4)	9 (81.8)	43 (74.1)	52 (75.4)
**February 2022**	5 (10.0)	0	0	5 (8.6)	5 (7.2)
**TOTAL (row %)**	**50 (72.5)**	**19 (27.5)**	**11 (15.9)**	**58 (84.1)**	**69 (100)**

**Table 4:** Univariate and multivariable logistic regression analysis investigating factors associated with occupational COVID-19 infection. Results expressed as odds ratio unadjusted (OR) and adjusted (aOR), with 95%CI confidence intervals (CI 95%). Significant estimates reported in bold.

FACTORS	STRATA		UNIVARIABLE		MULTIVARIABLE MODEL I (1496 Complete Observations)		MULTIVARIABLE MODEL II (1503 Complete Observations)	
			OR (95%CI)	*p*-Value	aOR (95%CI)	*p*-Value	aOR (95%CI)	*p*-Value
**Age (years)**	**Linear term**		1.01 (1.00; 1.02)	0.105	0.99 (0.98; 1.01)	0.504		
**Sex**	**Females**		*reference*		*reference*			
	**Males**		0.89 (0.70; 1.15)	0.392	1.04 (0.75; 1.45)	0.811		
**COVID-19** **WAVES**	**II (Oct–Dec 2020)**		*reference*		*reference*		*reference*	
	**III (Jan–Mar 2021)**		1.01 (0.70; 1.45)	0.972	0.88 (0.56; 1.38)	0.570	0.88 (0.55; 1.36)	0.531
	**III b (Apr–Sept 2021)**		**0.30 (0.16; 0.54)**	**<0.001**	**0.30 (0.14; 0.65)**	**0.002**	**0.28 (0.18; 0.41)**	**<0.001**
	**IV (Oct–Dec 2021)**		**0.30 (0.21; 0.42)**	**<0.001**	**0.27 (0.18; 0.41)**	**0.000**	**0.30 (0.14; 0.64)**	**0.002**
	**V (Jan 2022)**		**0.13 (0.09; 0.18)**	**<0.001**	**0.09 (0.06; 0.13)**	**0.000**	**0.09 (0.06; 0.13)**	**<0.001**
**WORKSITE**	Non-clinical workers *		*reference*		*reference*		*reference*	
	**Administrative services**		**8.21 (2.14; 31.57)**	**<0.001**	**6.67 (1.05; 42.42)**	**0.044**	**3.50 (0.69; 17.7)**	**0.131**
	**Radiology & other hospital services**		**13.51 (3.93; 46.39)**	**<0.001**	**9.20 (1.56; 54.37)**	**0.014**	**4.93 (1.08; 22.58)**	**0.040**
	**Surgical wards**		**17.77 (5.40; 58.46)**	**<0.001**	**8.39 (1.46; 47.98)**	**0.017**	**4.76 (1.07; 21.06)**	**0.040**
	**Territorial health services ^$^/health care management**		**24.28 (7.52; 78.40)**	**<0.001**	**11.5 (2.07; 63.57)**	**0.005**	**6.25 (1.46; 26.79)**	**0.013**
	**Medical wards**		**36.53 (11.28; 118.25)**	**<0.001**	**19.51 (3.44; 110.49)**	**0.001**	**10.91 (2.49; 47.75)**	**0.001**
	**COVID-19 hospital units**		**134.56 (41.35; 437.88)**	**<0.001**	**62.21 (10.91; 354.94)**	**<0.001**	**34.95 (7.92; 154.25**	**<0.001**
	**A&E**		**210.96 (63.66; 669.06)**	**<0.001**	**132.27 (22.99; 760.98)**	**<0.001**	**74.56 (16.75; 331.06)**	**<0.001**
	**Academic staff/tutors**		NA		NA		NA	
**JOB TASK**	**Non-clinical** **workers**	**Blue Collars**	*reference*		*reference*		*reference*	
		**Clerks**	5.21 (1.44; 18.88)	**0.012**	*1.56 (0.29; 8.27)*	0.601	*2.2 (0.42; 11.59)*	0.349
		**Academic staff/tutors**	NA		NA		NA	
	**Clinicians**	Health technicians **	**8.93 (2.66; 29.97)**	**<0.001**	2.55 (0.51; 12.89)	0.256	2.55 (0.51; 12.89)	0.256
		**Physicians**	**12.78 (3.96; 41.27)**	**<0.001**	1.91 (0.39; 9.35)	0.424	2.73 (0.57; 13.15)	0.211
		**Nurses**	**27.38 (8.60; 87.14)**	**<0.001**	3.54 (0.73; 17.06)	0.115	**4.98 (1.25; 23.60)**	**0.043**
		**Nurse aides**	**35.66 (10.98; 115.87)**	**<0.001**	4.64 (0.94; 23.03)	0.060	**6.38 (1.31; 31.11)**	**0.022**
		**Students/postgraduate trainees**	NA		NA		NA	

* Primary care services, health districts; outpatient; public health department. ** Clerks, general cleaners, food workers, gardeners, maintainers, guardians, other ^$^ Laboratory services, radiology service.

## Data Availability

Data generated and analyzed during the current study are not publicly available, since they were purposively collected by the authors for the present study, but are available from the corresponding author on reasonable request.

## Supplementary Materials

The following are available online at https://www.mdpi.com/article/10.3390/v14081663/s1, File S1: Contact tracing questionnaire for SARS-CoV-2 infections.
