# Supplementary material for: SARS-CoV-2 Infection in Health Care Workers of Trieste (North-Eastern Italy), 1 October 2020–7 February 2022: Occupational Risk and the Impact of the Omicron Variant
Source: Viruses. 2022 Jul 28;14(8):1663. doi: 10.3390/v14081663 (PMC9413002; doi:10.3390/v14081663)
Supplement: Supplementary file 1 [file viruses-14-01663-s001.zip › viruses-1826467-supplementary-file-S1.pdf]

ASUGI
Azienda Sanitaria Universitaria Giuliano Isontina
REGIONE AUTONOMA FRIULI VENEZIA GIULIA

OCCUPATIONAL MEDICINE UNIT

CONTACT TRACING QUESTIONNAIRE FOR SARS-CoV-2 INFECTIONS

- Name and surname

- Date of Birth

- Sex (M/F)

- Occupation (physician, nurse, nurse aid, health technicians, student, post-graduate medical trainees, blue collar, clerk, academic staff/ tutor)

- Department (Covid-19 ward, medical, surgical; accident & emergency service; radiology & other hospital services; territorial health services /health care management; administrative services; academic teaching/clinical instruction units)

- COVID-19 vaccination status: number of doses, date of vaccination

- Date of PCR test positive for SARS-CoV-2

- Any contact (in the previous 14 days) with:

1) COVID-19 patients (yes/no); with or without protective equipment (yes/no)
2) colleagues positive for SARS-CoV-2, in the workplace (yes/no)
3) close friends positive for SARS-CoV-2 (yes/no)
4) housemates or household members positive for SARS-CoV-2 (yes/no)

- COVID-19 Symptoms: no/yes (specify)
